# Cultural Consistency in Late-Life Declines in Positive Personality Traits

**DOI:** 10.1093/geroni/igab046.2156

**Published:** 2021-12-17

**Authors:** William Chopik

**Affiliations:** Michigan State University, East Lansing, Michigan, United States

## Abstract

Personality has elements of both stability and change across the adult lifespan. There has also been evidence for terminal decline—late-life decreases in positive psychological characteristics. However, many of these studies have examined these patterns in primarily Western populations. The current study examined the consistency of age differences in positive personality traits (i.e., character strengths) across cultures. I examined 2,895,051 participants ranging in age from 13 to 100 (Mage = 34.31; 65.3% women) from 90 different countries. I reproduced patterns of terminal decline across cultures. In addition to mean differences between cultures (e.g., focusing on the present is associated with more positive traits [Mr = .45]), cultural characteristics often moderated the effects of age on positive personality traits. For example, terminal decline was more dramatic among people from collectivistic cultures and flatter among people from individualistic cultures. Results will be discussed in the context of cultural variation developmental processes.

